# Complications of Brentuximab Therapy in Patients with Hodgkin’s Lymphoma and Concurrent Autoimmune Pathology—A Case Series

**DOI:** 10.3390/hematolrep16020030

**Published:** 2024-05-20

**Authors:** Oana Diana Preda, Sorina Bădeliță, Iulia Ursuleac, Ruxandra Maria Irimia, Sonia Balanica, Monica Cojocaru, Cristina Cotruta, Camelia Dobrea, Daniel Coriu

**Affiliations:** 1 University of Medicine and Pharmacy “Carol Davila”, 050474 Bucharest, Romania; sorinabadelita@gmail.com (S.B.); ruxandramariairimia@gmail.com (R.M.I.);; 2 Fundeni Clinical Institute, 022328 Bucharest, Romania

**Keywords:** brentuximab vedotin, Hodgkin’s lymphoma, autoimmune disorders, multidisciplinary care, T-cell-mediated immunity, immunosuppression, dose reduction

## Abstract

**Background**: Brentuximab Vedotin (BV) has revolutionized the treatment landscape for Hodgkin’s lymphoma, yet its effects on pre-existing autoimmune disorders remain elusive. **Methods**: Here, we present four cases of patients with concurrent autoimmune conditions—Crohn’s disease, vitiligo, type I diabetes, and minimal change disease—undergoing BV therapy for Hodgkin’s lymphoma. The patients were treated with A-AVD instead of ABVD due to advanced-stage disease with high IPI scores. **Results:** Our findings reveal the surprising and complex interplay between BV exposure and autoimmune manifestations, highlighting the need for multidisciplinary collaboration in patient management. Notably, the exacerbation of autoimmune symptoms was observed in the first three cases where T-cell-mediated autoimmunity predominated. Additionally, BV exposure precipitated autoimmune thrombocytopenia in the vitiligo patient, underscoring the profound disruptions in immune regulation. Conversely, in the minimal change disease case, a disease characterized by a blend of B- and T-cell-mediated immunity, the outcome was favorable. **Conclusions**: This paper underscores the critical importance of vigilance toward autoimmune flare-ups induced by BV in patients with concurrent autoimmune conditions, offering insights for tailored patient care.

## 1. Introduction

Classical Hodgkin’s lymphoma (HL) is a relatively rare malignancy arising from post-germinal center B lymphocytes that have lost their characteristic gene expression profile. The resulting malignant cells, also known as Reed–Sternberg cells, harbor CD15 and CD30 and can be found in a relatively small number in the tumoral environment, surrounded by a plethora of immune cells [1]. The 5-year survival rate can reach around 90% in localized cases, and the majority of patients are now cured with front-line therapy [2]. One of the factors associated with these improved outcomes is the introduction of the antibody–drug conjugate Brentuximab Vedotin (BV). BV consists of a chimeric monoclonal antibody directed against CD30, conjugated with the antimitotic agent monomethyl auristatin E. The efficacy of BV was established by a phase III randomized trial in newly diagnosed HL patients, as well as in a relapsed or refractory setting [3].

Autoimmune diseases (ADs) arise as a result of an abnormal immune reaction to self-antigens, leading to the destruction of various organs and tissues [4]. The etiopathogenetic mechanism behind the aberrant immune targeting of self-cells is considered to be a result of complex interactions between genetic predisposition and environmental triggers. The complex associations between AD and lymphoproliferative diseases have long been extensively described [4,5]. Based on the cell lineage that is predominantly involved in the process, autoimmune conditions have been divided into B-cell mediated (such as myasthenia gravis, rheumatoid arthritis, systemic erythematous lupus, etc.) and T-cell mediated (inflammatory bowel disease, multiple sclerosis, type 1 diabetes, etc.) [6,7,8,9,10,11,12].

The role of CD30, also known as Tumor Necrosis Factor Receptor Superfamily Member 8, in autoimmune disease is still not fully characterized. It can vary depending on the specific autoimmune disease, the context of its expression, and the interplay with other immune molecules [13]. However, several possible mechanisms link CD30 to the development of AD. Generally, CD30 is naturally expressed on activated B and T lymphocytes, as well as NK cells, monocytes, macrophages and eosinophils. The levels are particularly high on allo-reactive and regulatory T cells. CD30 is also involved in the expansion of memory T cells via the interaction with CD153 [14]. High levels of CD30 both in the serum as well as on activated immune cells have been described in an array of autoimmune conditions including atopic dermatitis, asthma, multiple sclerosis, Systemic Lupus Erythematosus, Wagener’s granulomatosis and Hashimoto Thyroiditis [15,16]

Depending on the cell types and costimulatory signals involved, CD30-mediated signal transduction events are capable of promoting cell proliferation, cell survival or anti-proliferative effects and cell death [17,18].

The molecular mechanism underlying such differential responses of different tumor cell types to anti-CD30 antibodies is not completely understood. The role of BV in the treatment of autoimmune conditions such as multiple sclerosis has been explored; however, few papers have reported the emergence of autoimmune conditions in lymphoma patients treated with BV in recent years [19,20]. A recent study has shown that in preclinical T-cell-activation models, BV can selectively deplete CD30-expressing Tregs, amplifying cytotoxic CD8 T-cell expansion. This finding can suggest that in patients with pre-existing autoimmune conditions that are T-cell mediated, exposure to BV can possibly lead to an exacerbation of the disease.

To this end, the administration of BV in Hodgkin patients associated with concurrent autoimmune conditions represents a therapeutic challenge.

We present a case series of four advanced Hodgkin’s lymphoma patients associated with classical T-cell-mediated AD, treated with Brentuximab Vedotin (detailed in Table 1).

## 2. Case I: Crohn’s Disease

Crohn’s disease (CD), together with Ulcerative Colitis (UC), are recognized as the main two representatives of the inflammatory bowel disease spectrum [21]. As a general definition, these diseases are considered to develop in susceptible individuals as a result of the complex interactions between environmental factors, microbiome, and an abnormal immune system. The microbiome appears to be an essential element of the etiopathogenesis of CD, since in the absence of enteral bacteria, it is impossible to recreate the inflammatory changes in animal models. 

Although in the beginning, the proposed mechanism for inflammatory lesions was based on the production of allo-reactive antibodies, it was later recognized that the T-cell-mediated autoimmunity represents the center of the etiopathogenetic process in CD [22]. In particular, the abnormal production of proinflammatory cytokines, INF-γ and transforming growth factor (TGF)-β appears to play an essential role in the induction and perpetuation of the inflammatory process [23].

Brentuximab-induced colitis, including one case of apoptotic colitis, has been reported in a few isolated cases. However, it is still unclear and unreported how the therapy impacts the clinical course of pre-existing CD [24].

Case I: The first patient presented chronic diarrhea from the age of 17, but received a definitive diagnosis of ileal stenosing CD at the age of 24 years. He has no other relevant family or personal medical history. At the time of diagnosis, the ileo-colonoscopy showed a normal appearance of the colon, but with profound longitudinal ulcers, edema and erythema of the terminal ileum; multiple biopsies were taken from the lesions, as well as from the colonic segments and rectum; all biopsies were consistent with ileal Crohn’s disease (the biopsies from the colon and rectum were normal). Subsequent chronic therapy with Budesonide was initiated.

In December 2021, 2 months after CD diagnosis, the patient was referred to our clinic for stage IVB mixed-cellularity classical Hodgkin’s lymphoma, and A-AVD therapy in standard doses was started. After three cycles of therapy, the PET-CT was consistent with a complete metabolic response, and the colonic biopsy showed histologically inactive CD. 

After the fifth complete cycle of A-AVD, the evolution of CD worsened, with an increase in the number of daily stools (up to six bloody stools/day), and the C-reactive protein level soared. After careful consultation with the gastroenterologist, we decided to continue the BV-based lymphoma therapy up to the sixth cycle. A change in the gastroenterological treatment was planned upon the completion of hematological therapy. 

At the end of six cycles of A-AVD, the PET-CT was consistent with a complete metabolic response of the lymphoma. However, the terminal ileal loop showed marked thickening of the wall and inflammatory enlarged adjacent lymph nodes. We consulted again with the gastroenterologist and decided to initiate the anti-α_4_β_7_ integrin antibody, Vedolizumab, and re-evaluate the patient using a PET-CT scan every 6 months. Anti-TNF alpha therapy could not be taken into account due to the pre-existence of hematological malignancy. The initial evolution under Vedolizumb seemed slowly favorable, although without clear remission of the inflammatory changes; however, due to the lack of suitable options and the seemingly stationary evolution, we decided to continue the therapy with close monitoring. The evolution of the patient slowly improved.

Six months after the completion of A-AVD, in February 2023, the patient relapsed with mediastinal bulky disease and further required treatment with standard chemotherapy (IGEV). Subsequently, in May 2023, the patient suddenly developed fever, nausea, diffuse abdominal pain and intense asthenia and was hospitalized in the gastroenterology department, where our colleagues performed an abdominal CT scan which showed multiple abscesses in the right flank, pelvis and perihepatic, for which evacuatory laparotomy with drainage and terminal ileal enterotomy was required; the postoperative evolution was favorable. Our colleagues considered that the patient was a secondary non-responder to this therapy, so the treatment was completely stopped after the surgery. In July 2023, the patient underwent autologous stem-cell transplantation. The subsequent PET-CT was consistent with a complete metabolic response and the patient was initiated on Brentuximab maintenance according to EBMT and NCCN Guidelines, due to the increased risk of relapse (I relapse under 12 months). Starting from January 2024, the patient started receiving Ustekinumab and the clinical evolution is favorable.

## 3. Case II: Vitiligo

Vitiligo is a relatively common autoimmune disease, characterized by the destruction of melanocytes leading to disfigurative hypopigmentation. Although certain genetic variants have been associated with predisposition towards the development of the disease, supported by a decreased ability to reduce oxidative stress, the definitive mechanism behind the destruction of the melanocytes is the aberrant function of CD8+ T cells. Among the traces associated with genetic predisposition are the errors at residue 135 for HLA-DQB1 and 45–46 for HLA-B and alterations in the FOXD3, PDGFRA, PTPN22 or IKZF4 gene [24,25].

The activity of CD8+ cytotoxic T cells is mandatory for disease onset and maintenance. In affected individuals, skin biopsies reflect a dense infiltrate of cytotoxic T cells in the hypopigmented areas. The chemotaxis of cytotoxic T cells towards the non-vascularized epidermis is led by IFN-γ and IFN-γ-induced chemokines. While the prime destruction is induced by effector T cells, the perpetuation of the aggression against melanocytes is maintained by resident memory T cells. This process is facilitated by the dysfunction of regulatory T cells and by the abnormal activation of the type 1 interferon (IFN) pathway.

One case report of Vogt–Koyanagi–Harada-like panuveitis has been described as a result of Brentuximab Vedotin exposure. This granulomatous inflammation of the uveal tract is considered to be T-cell mediated and is often associated with vitiligo. However, in the case report, the patient did not present with the typical vitiligo association, and so far, to our knowledge, this is the first report describing the clinical evolution of vitiligo lesions as a result of BV exposure.

Case II: Presenting with stage IV Hodgkin’s lymphoma and vitiligo, our second patient is a 54-year-old female, diagnosed from a young age with vitiligo. Her personal history is otherwise unremarkable; however, from her family history, we learned that her mother also had a diagnosis of vitiligo. In December 2021, she presented with a persistent cough, for which she initially received symptomatic treatment without improvement. Five months later, due to the symptoms’ persistence, she underwent a thoracic CT scan that showed a large tumoral mass in her right apical lobe, associated with multiple latero-cervical and mediastinal adenopathies. The biopsy of the tumoral mass was consistent with a diagnosis of classical Hodgkin’s lymphoma, the Nodular Sclerosis subtype. She was referred to our department in July 2022 and started Brentuximab–AVD in standard doses due to advanced disease and heavy smoking (40 packs-years). 

Upon the completion of two courses of therapy, the PET scan was consistent with a Deauville score of 2. No skin changes were observed during this initial period.

During the third cycle of treatment, she rapidly developed extensive hyperpigmented, non-pruriginous lesions on over 80% of the body surface, surrounding the previous hypopigmented lesions. After referral to the dermatology department, she initially received systemic treatment with Bilastinum and topical corticosteroids, and the Brentuximab dose was reduced to the “−1”, 0.9 mg/kg level. However, the patient showed no improvement, and she subsequently required a skin biopsy that showed nonspecific diffuse lymphocytic inflammatory infiltrate and vacuolization of the dermal–epidermal junction. Unfortunately, no additional immunohistochemical staining was performed to determine the exact nature of the lymphocytic inflammatory infiltrate.

Interestingly, after the fifth course of therapy, she developed grade III thrombocytopenia. The bone marrow aspirate was consistent with a megakaryocytic hyperplasia, supporting a diagnosis of immune thrombocytopenia, probably in the context of Brentuximab therapy.

**Figure 1:**
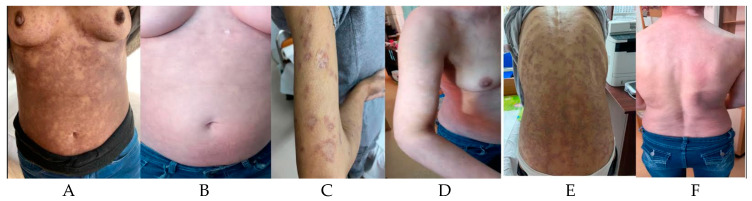
Anterior thoracic aspect during treatment (**A**) and after treatment (**B**); Upper limb aspect during treatment (**C**) and after treatment (**D**); Posterior thoracic view during treatment (**E**) and after treatment (**F**).

After the complete course of six cycles of therapy, the patient maintained a complete metabolic response. After ending the therapy, the skin lesions slowly started to improve. Six months after the completion of therapy, the patient maintains the hematological response and shows a complete resolution of the hyperpigmented skin lesions (Figure 1).

## 4. Case III: Type I Diabetes

Type I diabetes is an autoimmune disease with a strong genetic predisposition, leading to the destruction of the insulin-secreting pancreatic beta cells. Among the genetic factors, two chromosomal regions, including the HLA region at the short arm of chromosome 6, locus 6p21.3 and the insulin gene region at chromosome 11, locus 11p15, have been recognized to be associated with the development of type 1 diabetes. Among these, HLA type II variants, HLA-DQA1, DQB1 and DRB, have the strongest correlation with the development of the disease.

Eventually, the definitive effector of the immune destruction is considered to be the cytotoxic T lymphocyte. The simplified disease model implies that an initial lesion of the pancreatic beta cells determined by exposure to environmental or infectious factors (such as viruses) will lead to the release of beta cell antigens and the abnormal activation of the T cells against them.

Both clinical trials as well as real-life reports have documented instances of BV-associated hyperglycemia, including diabetic ketoacidosis and BV-induced diabetes. The reported risk of developing hyperglycemia in the existing clinical trials stands at 8%. In one report presenting the development of severe hyperglycemia and cytokine release syndrome in an HIV-associated HL, the authors hypothesize that the abnormal T-cell function resulting from HIV infection coupled with the potential attenuation of T-cell regulation induced by CD30 targeting by BV led to a severe autoimmune response against the insulin receptor. Moreover, all case reports consistently indicate elevated levels of C peptide, implying a potential association between BV-induced hyperglycemia/diabetes and the T-cell-mediated destruction of insulin receptors. However, the impact of BV therapy on a patient with pre-existing type I diabetes remains unexplored in the current literature.

Case III: Our third patient is a 44-year-old female, diagnosed with type I diabetes at the age of nine, and no other significant prior history except for being a heavy smoker (40 package years).

In February 2022, she was referred to our department for a diagnosis of classical stage IV B Hodgkin’s lymphoma with mixed cellularity and associated with liver involvement. She was initiated on Brentuximab–AVD therapy in standard doses. 

After the first dose of therapy, the blood sugar levels dramatically increased, reaching constant values of over 300 mg/dL throughout the six courses of therapy treatment. Despite cooperating closely with the diabetologist and modulating the insulin dose, the glycemic values remained high. The patient did not receive corticosteroid premedication with antiemetics due to the existing diabetes.

In addition, after the second dose of A-AVD, the patient developed a generalized skin rash that was interpreted at the time as an allergic reaction to sulphamethoxazole and trimethoprim. Sulphamethoxazole and trimethoprim was discontinued, with the resolution of symptoms. 

**Figure 2:**
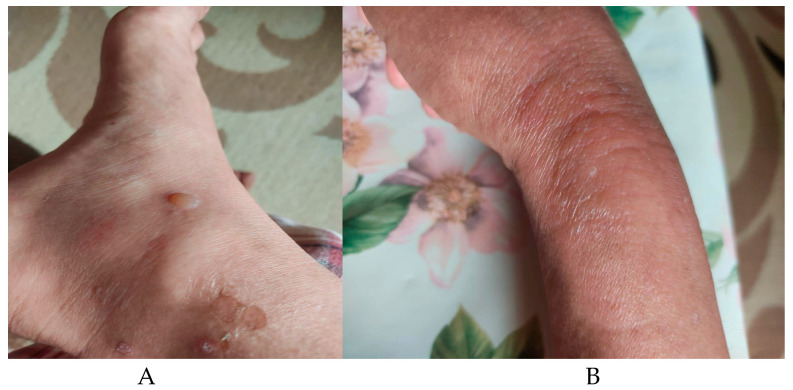
Bullous lesions in Type I Diabetes. Ankle (**A**) and foot (**B**) bullous lesions and erythema.

However, during the sixth cycle of therapy, the patient developed grade II peripheral neuropathy with pain and localized bullous vesicles on the hands and feet (Figure 2).

The dermatologist considered the lesions as an allergic reaction to the therapy which required topical and systemic corticosteroids, and the last dose of Brentuximab was omitted.

Upon the completion of therapy, the PET-CT was consistent with a complete metabolic response.

One month after the completion of therapy, her glycemia levels returned to the normal range and the insulin requirements dropped to the pre-treatment values. The neurological symptoms slowly abated during the next 3 months, while the skin rash remitted in the first weeks after the completion of therapy.

## 5. Case IV: Minimal Change Disease with Nephrotic Syndrome

Minimal change disease (MCD) is one of the most common causes of nephrotic syndrome in children, but it is also found in adults. The increased glomerular membrane permeability with subsequent albuminuria leads to decreased intravascular volume and peripheral edema [26].

MCD is considered to be an autoimmune disorder caused by a dual mechanism. The first step is considered to be aberrant CD80 expression by podocytes as a result of the exposure to circulating cytokines (Il-13), infectious agents (viral RNA (poly (I:C), lipopolysaccharides) or allergens. Subsequently, the pre-existing dysfunctional regulatory T cells are unable to secrete the CTLA-4, IL-10 and TGF-β required for the down-regulation of CD80 expression. As a result, CD80 binds to the CD28 expressed on the T cells, leading to T-cell activation. Also, the expression of CD80 on podocytes induces a conformational change, which increases glomerular permeability and proteinuria. Additional arguments that support the T-mediated theory of the etiopathology of the disease also include the high remission rates after measles infection, the absence of electron-dense membrane deposits, the common association with Hodgkin’s lymphoma and the response to suppressors of classical cell-mediated immunity [26].

Recent research has also unveiled the presence of auto-antibodies against nephrin in MCD, an important component of the slit diaphragm. However, the lack of electron-dense membranous deposits represents an aspect that still needs to be further investigated. 

The association between MCD and Hodgkin is widely recognized, and it is often considered a paraneoplastic manifestation. One article proposed the expression of c-mip by Reed–Sternberg cells as a possible cause for the association. This leads to further interaction with PAG and Fyn, which in turn shifts the T cells from a resting to an activated state.

However, to our knowledge, there are no reports on the effect of BV therapy on the evolution of the disease.

Case IV is a 29-year-old male, diagnosed at the age of 10 with MCD. He received, over the course of time, therapy with corticoids, cyclophosphamide and Rituximab. 

In August 2021, he was referred to our department for further diagnosis work-up due to the presence of a latero-cervical and a mediastinal mass. The immunohistochemical diagnosis was classical Hodgkin’s lymphoma, rich in lymphocytes. The initial PET-CT staging was IV due to hepatic involvement. 

At the moment of diagnosis, he was receiving nephrological immunosuppressive treatment with cyclophosphamide and mycophenolate mofetil. After complete diagnosis work-up and careful consultation with the nephrology department, we commenced therapy with Brentuximab–AVD in standard due to advanced disease and a high IPI score. At that moment, the proteinuria level was 6.82 g/24 h, with a creatinine level of 1.48 mg/dL and a creatinine clearance of 78 mL/min EGFR-CKD EPI. We maintained only a low dose of mycophenolate mofetil due the persistence of massive proteinuria.

After the first dose of A-AVD, the patient presented with profound pancytopenia and creatinine increase (2.13 mg/dL; urea 79.6 mg/dL), and after careful consultation, we decided to cease the nephrologic therapy. Subsequently, at the next visit, the creatinine levels dropped to the baseline values (1.3 mg/dL). 

The evolution was complicated by multiple septic adverse events, ileus, grade II peripheral neuropathy and severe taste perversion, requiring parenteral nutrition. Starting with the third cycle, the dose of Brentuximab was reduced to the level of “−1”, with improvement in the neurological symptoms but persistent septic events.

After six cycles of therapy, the patient achieved and maintained a complete metabolic response.

At the end of the treatment, the proteinuria was 0.64 g/24 h with a creatinine level of 0.9 mg/dL, and a creatinine clearance of 119 mg/dL EGFR-CKD EPI did not require further nephrological treatment.

## 6. Conclusions

Brentuximab Vedotin has been widely used in the past decade in the treatment of Hodgkin’s lymphoma and other CD30-expressing non-Hodgkin lymphomas. However, there is a gap of knowledge regarding the exact impact BV has on the natural history of previously existing T-cell-mediated autoimmune disorders.

We hereby present the cases of four patients with Crohn’s disease, vitiligo, Type I diabetes and minimal change disease who underwent treatment with BV for concurrent advanced Hodgkin’s lymphoma and reflect the intricate and often unexpected effects of BV exposure on autoimmune manifestations. All cases were young patients with advanced-stage disease and high IPS scores, and we decided to treat them with A-AVD to increase the response rate, OS and PFS, which is a decision supported by the Echelon-1 study. All the cases presented with a high degree of complexity and required a multidisciplinary team for management.

Interestingly, in the first three cases, where the mechanism of autoimmunity is being clearly recognized as T-cell mediated, the clinical evolution significantly worsened during the treatment with BV. This can be possibly explained by the previously described Tregs depletion associated with the amplification of cytotoxic CD8 T-cell expansion.

Interestingly, the vitiligo patient additionally developed autoimmune thrombocytopenia as a result of BV exposure, suggesting clear disruptions of the immune regulation.

In the fourth case, describing a case of minimal change disease, a condition that results from a blend between B- and T-cell-mediated immunity, the evolution of the patient was favorable after the cessation of immunosuppression with mycophenolate mofetil. This agent impacts both B and T cells. We can only speculate that BV exposure resulted in a depletion of activated B cells and the reduction in auto-antibodies against nephrin; however, the exact immunological mechanism remains unknown.

In conclusion, when managing patients with Hodgkin’s lymphoma and concurrent autoimmune conditions, it is of extreme importance to maintain a high degree of awareness regarding possible autoimmune flare-ups. Close consultation as part of a multidisciplinary team is essential for the successful management of these patients. Dose reductions in BV should be considered not only as a result of classically cited adverse events, but also in the setting of an exacerbated previous autoimmune condition.

Further research endeavors are warranted to unravel the intricate immunological mechanisms underlying BV-induced effects on autoimmune disorders and to refine therapeutic strategies tailored to the unique needs of these patients.

## Figures and Tables

**Table 1:** Patients characteristics.

Case Number	1	2	3	4
Age at diagnosis (years)	24	54	42	29
Sex	M	F	F	M
HL subtype	Mixed cellularity	Nodular Sclerosis	Mixed cellularity	Lymphocyte rich
Stage	IV B	IV B	IV B	IV B
Associated AD disease	Crohn’s disease	Vitiligo	Type 1 diabetes	Minimal change disease with nephrotic syndrome
Cell-lineage mediator	T-cell mediated	T-cell mediated	T-cell mediated	T/B-cell mediated
Treatment	A-AVD	A-AVD	A-AVD	A-AVD
HL outcome	CR	CR	CR	CR
AD outcome	Worsened	Worsened	Worsened	Improved
Number of cycles until progression of the autoimmune disease	5	2	1	N/A

M-male, F-female, HD-Hodgkin Lymphoma type, IVB-stage IVB, AD-advanced disease, CR-complete remision, N/A-not aplicable.

## Data Availability

The data presented in this study can be provided upon reasonably request.

## List of Abbreviation

BV: Brentuximab Vedotin, HL: Hodgkin’s lymphoma, AD: autoimmune disease, MCD: minimal change disease, EBMT: European Society for Blood and Marrow Transplantation, NCCN: National Comprehensive Cancer Network, A AVD Protocol: Brentuximab Vedotin, Doxorubicin, Vinblastin, Dacarbazine.
